# Universal versus tailored solutions for alleviating disruptive behavior in hospitals

**DOI:** 10.1186/s13584-015-0018-7

**Published:** 2015-09-01

**Authors:** Talia Berman-Kishony, Shifra Shvarts

**Affiliations:** Ombudsperson, Department of Management, Rambam Medical Center, Haifa, Israel; Visiting Research Fellow, Harvard Medical School and Beth Israel Deaconess Medical Center, Boston, MA USA; Center for Medical Education, Ben-Gurion University of the Negev, Beer Sheva, 84105 Israel

**Keywords:** Nurse-physician conflicts, Disruptive behavior, Conflict resolution, Patient satisfaction, Quality of care, Patient safety

## Abstract

**Background:**

Disruptive behavior among hospital staff can negatively affect quality of care. Motivated by a standard on disruptive behavior issued by The Joint Commission (LD 3.10), as well as the desire to improve patient care, minimize liability, and improve staff retention, hospitals are setting policies to prevent and resolve disruptive behaviors. However, it is unknown whether uniform conflict management tools are equally effective among different hospital settings.

**Methods:**

We surveyed residents and nurses to identify similarities and differences among hospital departments in the antecedents, characteristics, and outcomes of disruptive behaviors, and in the effectiveness of conflict management tools. We used a quantitative questionnaire-based assessment to examine conflict perceptions in eight different hospital departments at Rambam Medical Center in Haifa, Israel.

**Results:**

Most participants (89 %) reported witnessing disruptive behavior either directly or in other parties; the most significant causes were identified as intense work, miscommunication, and problematic personalities. The forms of these behaviors, however, varied significantly between departments, with some more prone to expressed conflicts, while others were characterized by hidden disruptive behaviors. These outcomes were correlated by the antecedents to disruptive behavior, which in turn affected the effectiveness of alleviating strategies and tools. Some tools, such as processes for evaluating complaints, teamwork and conflict management courses, and introducing a behavioral mission statement, are effective across many antecedents. Other tools, however, are antecedent-specific, falling into two principal categories: tools directly removing a specific problem and tools that offer a way to circumvent the problem.

**Conclusions:**

Conflict resolution tools and strategies, based on residents and nurse perceptions, may be more effective if tailored to the specific situation, rather than using a “one-size-fits-all” approach.

**Electronic supplementary material:**

The online version of this article (doi:10.1186/s13584-015-0018-7) contains supplementary material, which is available to authorized users.

## Background

The relationship between residents and nurses is known as an area of endemic conflict [1–8]. Disruptive behaviors among healthcare personnel range from subtle questioning of judgment through explicit threatening behaviors to physical insults [9–13]. Such behaviors negatively impact staff relationships, communication efficacy, and, most critically, patient care and clinical outcomes [4, 6].

Alerted by the accumulating evidence implicating disruptive behavior in hurting staff morale and endangering patients, the Joint Commission issued a sentinel event alert that recommends developing and implementing policies that address and alleviate disruptive behaviors [12]. However, while various strategies and tools have been developed, little is known about their effectiveness and how to best tailor specific strategies and tools to specific antecedents and forms of the conflicts. Our goal was to study whether some strategies are more effective in specific departmental settings or circumstances and to learn if their effectiveness depends on unique antecedents contributing to disruptive behavior.

Antecedents of conflicts between nurses and physicians are comprised of personal, interpersonal, and organizational factors. Personal factors occur due to personality and attitude differences between people [7, 14], as well as education [1, 15], age, generational diversity [16], gender [17] and values [14, 18, 19].

Interpersonal factors contributing to conflicts include interpersonal incompatibilities [20], distrust [21], disrespect [22, 23] and poor communication styles [24, 25]. Conflicts initiated by interpersonal antecedents are typically counterproductive, focusing on personal antagonism rather than on specific issues related to the organizational function that need to be resolved [19, 26].

Organizational factors arise due to differing viewpoints and opinions regarding the overall goals and content of a team’s task. Issues such as lack of alignment of incentives, competing priorities, and ambiguity in mission and objectives promote task conflict [27]. Interdependence [28, 29], power imbalances between disciplines [30], and the interaction between organizational leaders with employees all influence an organization’s culture [31] and contribute to the development of task conflicts. Task conflicts often lead to frustration [32], though it can also be constructive in terms of promoting team working and productivity [33].

All of these antecedents - personal, interpersonal and organizational - influence relationships between nurses and physicians. Based on differences in occupations and roles, gender and culture, intense work and short staffing, conflicts between residents and nurses are inevitable [34]. Nurses usually have significantly more experience compared to residents, but residents often outrank nurses in decision-making authority [16, 35]. The gender issue, though in a state of transition, is also relevant [17].

Disruptive behavior can take a spectrum of forms and be either overtly expressed or remain hidden [36]. Common types of disruptive behaviors include impatience with questions [37], failure to respond to phone calls and pages [9], verbal abuse and patronizing language [38–40], disrespect for others, especially with less power [10, 41], and threatening body language and physical abuse [42].

Many strategies and tools to alleviate disruptive behavior have been suggested, implemented, and tested [37, 43]. These strategies and tools range in their level of formality. Formal strategies and tools include professional conduct policies [44–47], dispute resolution mechanisms [48–50] and provision of education and training programs on conflict management skills, and team training [51–56]. Less-formal opportunity-generating mechanisms include multidisciplinary meetings [57–59], joint nurse-physician “intentional” rounds at the bedside [60] video rounding collaboration [61] and personal intervention by organizational leaders [42, 62]. While studies have tested and shown the effectiveness of these various tools, it remains unclear whether and how tool effectiveness varies based on conflict causes and patterns.

We analyzed questionnaire responses of nurses and physicians in different hospital departments to find correlations between conflict antecedents and forms to the perceived effectiveness of different conflict alleviating tools. Following [42] and [6], our questionnaire was designed to probe characteristics, antecedents, consequences and strategies to address disruptive behaviors, but here we also added the correlation of strategies and tools to conflicts forms and antecedents. The analysis presented follows a dissertation work by TBK [63], which incorporated also a qualitative analysis of disruptive behavior at different hospitals. Thorough analysis of questionnaire responses was aimed at quantifying: (1) differences in conflict antecedents and patterns between hospital departments; (2) differences and commonalities of the effects of conflict on staff and patients in different hospital departments; and (3) effectiveness of specific conflict management tools, with particular attention at their dependence on conflict antecedents. By analyzing the questionnaire data for correlations among responses, we were able to examine whether effective solutions for some settings might be deemed ineffective in other settings.

## Methods

### Questionnaire

A questionnaire was designed to assess the forms, antecedents, consequences and solutions to conflicts between residents and nurses in different hospital departments (See Additional file 1). To help design the questionnaire a preliminary work was conducted via focus group discussions with nurses and residents at two hospital departments (labor and delivery and anesthesia) at a local Boston hospital to explore the main themes of disruptive behavior. Focus group discussions, in three different hospital environments helped us get a more comprehensive understanding of disruptive behavior phenomena - its antecedents, consequences and potential outcomes. The meetings provided also the opportunity to validate the questionnaire in terms of verbal, content and cognitive understanding.

Based on existing surveys [6, 42] and following the preliminary findings related to disruptive behavior themes, we formulated the questionnaire and performed verbal, cognitive and content validation through focus group discussions with nurses and residents in three different hospital departments at Sorokah Medical Center (Anesthesia, Labor and Delivery, and Pediatric Intensive Care). Additional input was provided by the hospital’s management and supplemented by in- depth interviews with stakeholders including nurses, residents and department leaders at Rambam Medical Center. The questionnaire consisted of 41 multiple-choice and yes or no questions; 5 point scale statements, ranging from 1 (low) to 5 (high). The questionnaire probed four main attributes of disruptive behavior: forms, causes, effects and tools for managing and alleviating conflict. Forms of disruptive behavior included a spectrum of behaviors: from refusing to speak or work with colleagues, to inappropriate joking, to yelling and cursing. Causes of disruptive behavior included personal, interpersonal, and organizational factors. Effects of disruptive behavior included the consequences on staff morale, patient safety and quality of care. Strategies and tools for alleviating disruptive behaviors included a variety of options aimed at addressing personal, interpersonal and organizational sources of disruptive behavior. Demographic data included gender and age of respondent, role/job, years of professional experience and the name of their hospital department.

### Settings

Rambam Medical Center in Haifa Israel is the largest public hospital in the Northern part of Israel and provides medical care to over two million patients. Rambam’s Emergency Department and Trauma units are among the most active in Israel and Rambam provides the full spectrum of healthcare services. Rambam Medical Center contains around 1000 inpatient beds for both children and adults patients.

Being the largest hospital in the northern part of Israel, Clinical activities include 81,610 hospitalizations and 602,859 outpatient visits annually.

To learn about the patterns related to disruptive behaviors, we conducted a comparative quantitative study of nurse-physician perceptions of conflicts in eight different departments (Dermatology; Neurology; Pediatric Intensive Care, ICU; Emergency Care, EC; Emergency Care Unit, ECU; Anesthesia; Surgery; Labor and Delivery), suggested by hospital management, at Rambam Medical Center. In choosing the departments involved we tried to capture variability in nature of departments, variability in size and differences in objective assessment of stress and pressure in departments. Accordingly, we selected small relatively calm department (dermatology) versus large and very busy department (emergency department).

Questionnaires were distributed to nurses and residents in all eight departments; a total of 134 respondents were analyzed (76 nurses and 58 physicians). The questionnaire was distributed in physicians and nurses separate meetings and was collected by the researcher, at the end of the meetings. Participation was fully voluntary and to avoid pressure on participants, no note was taken as to the number of people who chose not to participate.

Department representation is listed in Table 1.

**Table 1:** **Distribution of participants in the different departments**

**Department**	**Male**	**Female**	**Unspecified**	**Nurse**	**Physician**	**Total**
Anesthesia	15	3	1	0	19	19
Dermatology	3	11	0	5	9	14
ECU	3	14	1	17	1	18
ER	8	2	1	1	10	11
ICU	2	12	0	13	1	14
LD	10	15	0	9	16	25
Neurology	3	10	0	12	1	13
Surgery	3	16	1	19	1	20

### Quantitative analysis of questionnaire data

All questionnaire data was subsequently analyzed in Matlab and STATA. Data was analyzed for average responses, for each question for the entire cohort population as well as for each department. P-Values for correlation between demographic attributes and responses were analyzed using the Regress procedure (“glmfit.m” and “linhyptest.m” and STATA regress). Correlation matrix across variables was calculated using Matlab’s “corrcoef.m” function.

## Results and discussion

### Frequent disruptive behavior, most commonly through verbal abuse

**Fig. 1:**
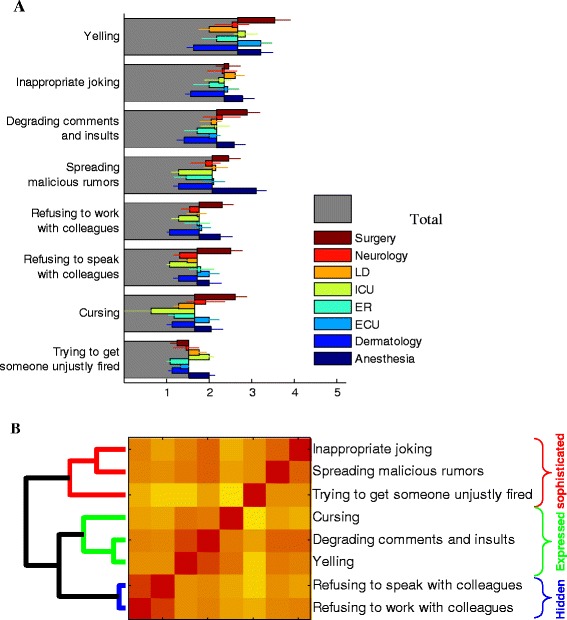
Forms of disruptive behavior. **a** Average scores of questionnaire on a scale of 1–5 for different forms of disruptive behaviors, for all participants (grey) and dissected by department (color, see legend; LD: Labor and Delivery, ICU: Intensive Care Unit, ER: Emergency Room, ECU: Emergency Care Unit). Error bars represnt standard error of the mean (standard deviation divided by square root of number of participants). **b** Clustering algorithm (left dendogram) applied to the correlation matrix between each two Forms of disruptive behavior (color-map represent the correlation; red – high correlation, yellow- low correlation) reveals natural grouping of the forms into ‘Hidden’, ‘Expressed’ and other more ‘Sophisticated’ forms. See text for more details

The frequency of disruptive behaviors varied substantially, with verbal abuse being the most prevalent (Fig. 1a). A large fraction of the respondents noted that they were witness to disruptive behavior (89 %). The most commonly reported form of disruptive behavior was yelling, followed by inappropriate joking and then, degrading comments and insults and spreading malicious rumors. Refusing to work and talk with colleagues was less frequent. Cursing and trying to get someone unjustly fired was reported to be rare.

We performed regression analysis of contribution of the demographic factors – gender, role, and department – to the scores for each question in the questionnaire. The model takes the binary variables of sex (0: Female, 1: Male) and role (0: Nurse; 1: Physician) as well as a binary variable for each department (0 if participant is not in the department, 1 if it is). Because the 8 variables of the department are dependent (participant always appear in one and only one department), we omit one of these variables and perform the regress with 7 variables representing the 8 departments (the 8^th^ variable is completely determined by the 7 variables). Role and Sex are also very highly correlated – The correlation coefficient is 0.57 with a P-value of 10^−12^ (most nurses are female and most physicians are males), further participants did not always indicate sex – therefore we chose to omit the sex variable and only looked at role which is highly correlated with sex. The scoring data was normalized for each participant between its minimal and maximal score to control for the fact that some participants always use the high (e.g., 4–5) scores while other use scores varying only at the low range of the scale.

**Fig. 2:**
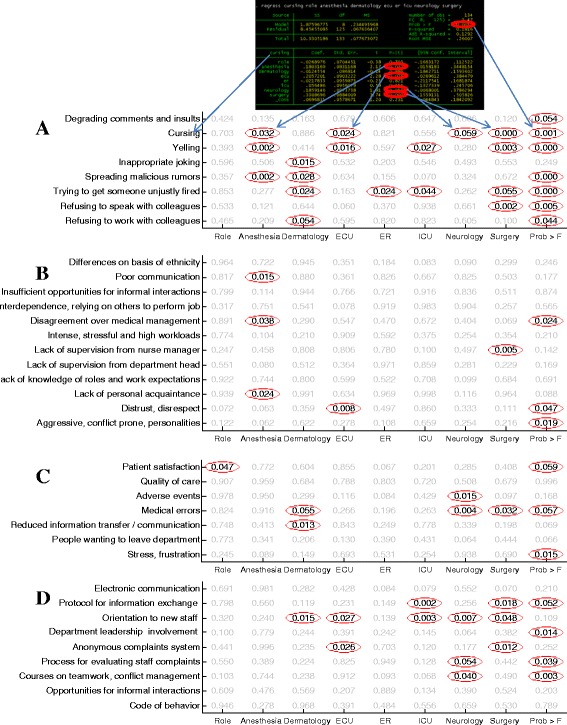
Significance of contribution of Role and Department to Forms **a**, Causes **b**, Consequences **c**, and Solutions **d** to disruptive behavior. Red ellipses point to significant P-values. The panel above explains how these results are derived from the STATA regress output (shown here for the case of “cursing”)

An example output of the REGRESS test for the case of the disruptive behavior “Cursing” is shown in Fig. 2. We performed this test for all of the questions in the questionnaire and recorded the corresponding P-Values for significance of the contribution of each of the demographic variables to the variance of the answers for each given question across participants of each (Fig. 2). The results show that role (and gender, done separately due to dependence between gender and role) had mostly non-significant contributions to participants’ answers for most of the questionnaire questions. In contrast, department affiliation had a significant correlation with participant answers to many of the questions, mostly with respect to Forms of disruptive behavior and also, though to less extent, the importance of the alleviating tools. We conclude that departments vary most significantly in the forms of disruptive behavior, and also show significant variations in the conceived importance of the proposed alleviating tools.

Department affiliation strongly affected the reported forms of disruptive behavior (Fig. 1a, colored bars). High variability was found between departments in the way people reported related forms of disruptive behavior. The Anesthesia, Surgery and Emergency Care departments were reported to “suffer” from significantly higher frequencies of almost all disruptive behavior forms, particularly vocal forms such as yelling and cursing as well as insults. Nurses and Residents in the Pediatric Intensive Care unit also reported high scores followed by Labor and Delivery where inappropriate joking received one of the highest scores relative to other departments. The Dermatology department, on the other hand, reported significantly less forms of disruptive behavior, especially the visible vocal forms.

The correlation among participants’ answers to different questions allows us to group the different Forms and Consequences of disruptive behavior into natural basic modes (Fig. 1b). Fig. 1b shows a matrix of pair-wise correlation between the participants’ answers to the different disruptive behavior forms. Participants tend to give similar scores to questions that they perceive as similar in nature. For example, “refusing to work with colleague” and “refusing to speak with colleague” get highly correlated responses (shown as red color in the matrix of Fig. 1b, indicating that participants gave correlated responses to these two questions). We can use these correlations to group the different questions into common classes. We have used a standard agglomerative hierarchical clustering algorithm (“linkage” in Matlab) [64]. This algorithm joins the most correlated items into groups and then keeps combining these groups into larger groups. At each step the most correlated groups or items are joined together. The result of the clustering is shown as a binary branching tree which shows how the individual items are joined hierarchically (see dendogram on the left of the matrix in Fig. 1b). Disruptive behavior forms clearly fell into Hidden (refusing to speak or talk with someone) and Expressed (Cursing, Yelling, Insults) classes as well as a separate class that included more sophisticated forms such as inappropriate joking and spreading malicious rumors.

### Personal, interpersonal and organizational factors contribute to disruptive behavior

**Fig. 3:**
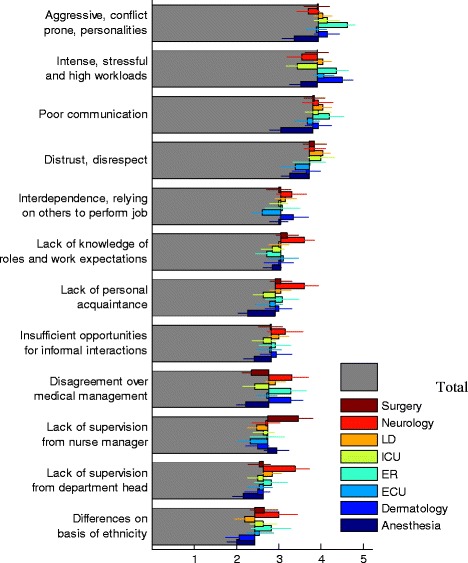
Factors causing disruptive behavior. Average scores of questionnaire on a scale of 1–5 for different Antecedents of disruptive behaviors, for all participants (grey) and dissected by department (color, see legend; LD: Labor and Delivery, ICU: Intensive Care Unit, ER: Emergency Room, ECU: Emergency Care Unit). Error bars are calculated as explained in Fig. 1

The highest reported causes for disruptive behavior were “aggressive conflict-prone personality”, “intense, stressful and high workloads”, “poor communication”, “distrust and disrespect” (Fig. 3). These high scoring factors represent personal, interpersonal and organizational factors, respectively. These results, therefore, rated the importance of personality of participants, as well as the work environment as structured by the organization and colleagues. At second, but still high level, we find the factors of interdependence, lack of professional and personal acquaintance, lack of supervision from the nurse manager and the department head, disagreements over medical management as well as insufficient opportunities for informal interactions to cause conflicts. Ethnicity difference was the least influential source of conflict.

We further examined differences in disruptive behavior antecedents based on department affiliation (Fig. 3, colored bars). Departments varied significantly in the antecedents of disruptive behavior in particular in aggressive conflict-prone personalities, distrust and disrespect and disagreements over medical management. In general, Anesthesia tended to give the lowest scores which was particularly interesting in light of the high rates of witnessing various forms of disruptive behaviors in Anesthesia (Fig. 1). For significance P-values see Fig. 2.

### Both patient and staff are negatively affected by disruptive behavior

**Fig. 4:**
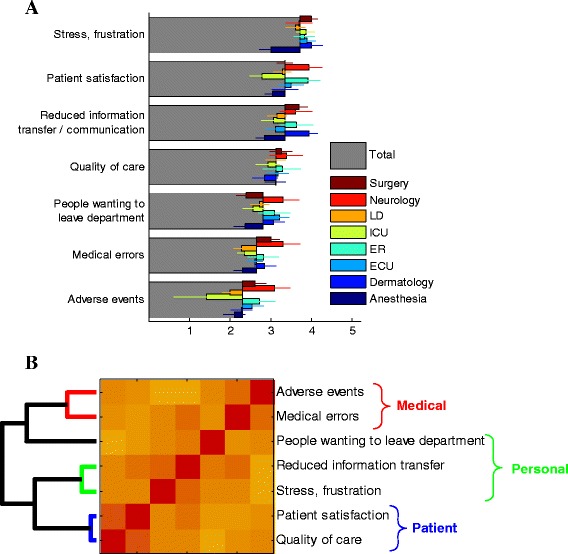
Consequences of disruptive behavior. **a** Average scores of questionnaire on a scale of 1–5 for different Consequences of disruptive behaviors, for all participants (grey) and dissected by department (color, see legend; LD: Labor and Delivery, ICU: Intensive Care Unit, ER: Emergency Room, ECU: Emergency Care Unit). Error bars are calculated as explained in Fig. 1. **b** Clustering algorithm (left dendogram) applied to the correlation matrix between each two Consequences of disruptive behavior (color-map represent the correlation; red – high correlation, yellow- low correlation) reveals natural grouping of the Consequences into ‘Medical’, ‘Personal’ and ‘Patient’ effects. See text for more details

Nurses and residents perceived that both patients and staff were negatively affected by disruptive behavior (Fig. 4a). The two most scored consequences of disruptive behavior represented a negative impact on both patients and staff. The first rated consequence was stress and frustration – which affected the staff. The second most commonly perceived consequence of disruptive behavior was a negative impact on patient satisfaction. The more severe results of disruptive behavior - medical mistakes and adverse events- appeared to be linked with disruptive behavior between nurses and physicians, but with a significantly lower frequency.

We found variability between hospital departments and consequences of disruptive behaviors (Fig. 4a, colored bars). Most affected by disruptive behavior were the Surgery and Neurology departments. Both had the highest incidences of clinically negative outcomes such as medical errors and adverse events. ER reported high effects on patient satisfaction. Dermatology, on the other hand, did not report much adverse events but did report higher than average reduction in information transfer. Anesthesia on the other hand had somewhat lower reported level of stress and frustration caused by disruptive behaviors. See Fig. 2 for significance values based on STATA regression analysis which accounts for department affiliation as well as the role of participants.

Similarly to the analysis of the Forms of disruptive behavior, here too we can use a clustering algorithm to automatically group the different questions according to correlations between participants’ responses (Fig. 4b). When two questions are perceived by the participants as similar, the response to these two questions tend to be correlated (high correlation is shown by more red colors in the matrix of Fig. 4b). The agglomerative hierarchical clustering algorithm revealed natural grouping of the Consequences of disruptive behavior by the subject of their effects. As can be seen in the figure, the naturally emerging groups are consequences related to the staff (people wanting to leave, impaired information transfer, stress and frustration), to the patients (satisfaction and quality of care) and to the medical treatment (medical errors and adverse events).

### Tools for alleviating disruptive behaviors must be tailored for specific conflict antecedents

**Fig. 5:**
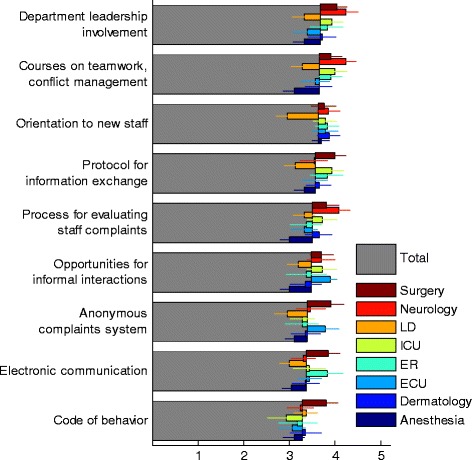
Importance of tools and mechanisms for alleviating conflicts. Average scores of questionnaire on a scale of 1–5 for different alleviating tools of disruptive behaviors, for all participants (grey) and dissected by department (color, see legend; LD: Labor and Delivery, ICU: Intensive Care Unit, ER: Emergency Room, ECU: Emergency Care Unit). Error bars are calculated as explained in Fig. 1

**Fig. 6:**
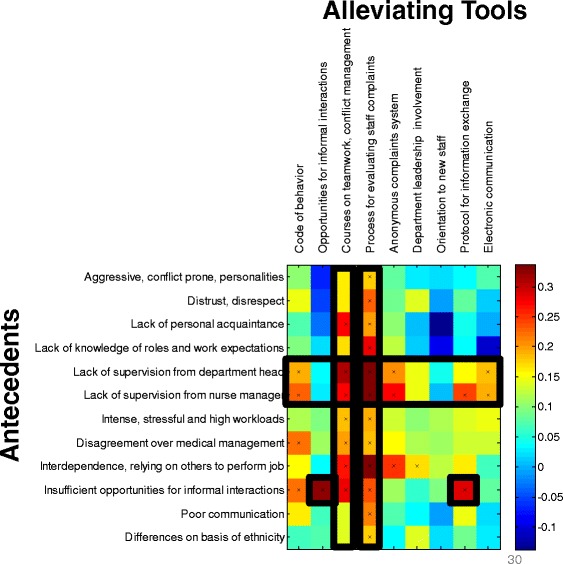
Correlation between effectiveness of Tools for alleviating disruptive behavior and the Antecedents of the conflict. Red color represents strong correlation between a specific antecedent and a specific tool for alleviating disruptive behavior. X’s signifies that the correlation is statistically significant (P value < 0.05). Some Tools are effective against many type of conflicts (vertical blocks), while others are effective only against specific type of conflicts (individually bolded squares)

All tools to address disruptive behavior were scored high with only mild variability in perceived effectiveness (Fig. 5). Participant responses regarding the effectiveness of potential solutions to alleviate disruptive behavior received uniformly high scores. To better understand possible differences in alleviating tools required for different circumstances, we analyzed the questionnaire data for correlations between suggested alleviating tools and the reported antecedents of disruptive behavior (Fig. 6). As seen in the figure, some tools are generally effective, while others are effective only for specific conflict antecedents. The generally effective tools include process for evaluating staff complaints and courses on teamwork and conflict management.

**Fig. 7:**
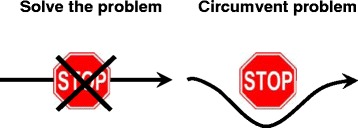
Two principal ways to resolve a given problem: either removing its antecedent or circumventing it

On the other hand, the effectiveness of other tools appears to critically depend on conflict antecedents. For example, in situations of “insufficient opportunities for interactions” a strong correlation was noted to the alleviating tool for generating “opportunities for informal interactions” and “protocol for information exchange”. These are two tools that directly address the lack of communication. The first correlated tool is a direct response to the alleviating tool while the second tool helps to circumvent the problem. Similarly, departments with lack of leadership, either by the department head or nurse manager required a collection of tools to substitute for this lack, such as “code of behavior”, or an “anonymous complaint system”. Another example is tools that generate information exchange such as “protocol for information exchange” and “electronic communication” which were found to be highly correlated with the antecedent of lack of leadership. Finally, a “code of conduct” was required in “disagreements over medical management” situations. Thus, these examples show that the certain alleviating tools must be prescribed based on the specific antecedents of the conflict. These antecedent-tailored tools resolve specific problems in two principal ways: directly - by removing the specific antecedent and indirectly - by circumventing the problem (Fig. 7).

There are universal intervention tools that are effective in every situation, such as teamwork education, value statements, and other general tools. However, there are other areas where tailored solutions are required to address the unique personal, interpersonal, and organizational issues affecting disruptive behavior.

Similarly to previous findings, our study shows high prevalence of disruptive behaviors in healthcare organization across all participating departments. As indicated, 89 % of respondents witnessed, either directly or in other parties, disruptive behaviors. Similarly, Rosenstein 2008 (sample size of 4350 at 102 VHA hospitals) showed a very high percentage of nurses and physicians experiencing disruptive behavior - almost 90 % of nurses witnessed disruptive physician behavior and about 70 % of physicians witnessed disruptive nurse behavior.

Similarly to the literature [6], conflicts negatively affect patient satisfaction, staff morale and wellbeing, as well as quality of care also at departments that are characterized to be less stressful and intense such as dermatology. Yet, we found variability between hospital departments in consequences of disruptive behaviors. Most affected by disruptive behavior were the Surgery and Neurology departments in terms of quality of care, providers’ well-being and patient satisfaction, while Anesthesia reported lower impacts of disruptive behaviors.

Supporting the literature, we found that disruptive behavior takes a spectrum of forms - from expressed confrontational verbal abuse such as yelling, cursing, insults [38, 39], disrespect for others [10, 41] towards hidden forms such as avoidance, “loud silence” lack of greetings or smiles [9]. We found that departments vary in expressed versus hidden conflicts. Some departments are more prone to verbal abuse (surgery, emergency room, anesthesia) and some to avoidance hidden forms (pediatric intensive care, dermatology).

Interestingly, our unsupervised analysis, were questions are grouped algorithmically based on correlation in participant responses, yielded classes of forms that match our understanding and preconception regarding the key modes of disruptive behavior. In particular, the forms of disruptive behavior clearly fell into three classes: Hidden Forms, such as refusing to speak or talk with someone, Expressed Forms such as Cursing, Yelling, Insults and a class that included more sophisticated forms such as inappropriate joking and spreading malicious rumors.

Whether hidden or expressed, our study shows that conflicts are common in all departments, also departments that are less exposed to visible forms of disruptive behaviors (such as dermatology).

Across departments, different combinations of personal, interpersonal and organizational factors lead to disruptive behaviors. It is possible that differences between departments are not only related to difference in their nature, tasks and levels of pressure, tension and intensity. For example surgery department compared to dermatology are very different in terms of the levels of stress. But also, the difference might relate to personality differences between surgeons and pediatricians. Personal factors vary substantially among departments; departments vary appreciably in personality of staff members and their leaders and conflict management style. Indeed studies have shown that single or just a few individuals with negative attitude can be directly or indirectly involved in the majority of conflicts in their department. Involvement of department heads and medical leadership is important in these circumstance as they are responsible and serve as role models in creating a safe culture and environment [62]. Interpersonal factors, including distrust, disrespect and miscommunication also vary among hospital departments. Finally, organizational factors, which include high levels of interdependence, power imbalance structures and hierarchies vary significantly.

Our study showed high correlation between effectiveness of tools and antecedents leading to disruptive behaviors. Looking at the correlation between antecedents and effectiveness of tools, we found that some tools are generally effective, while others are effective only for specific conflict antecedents. The generally effective tools include process for evaluating staff complaints and courses on teamwork and conflict management. On the other hand, the effectiveness of other tools appears to critically depend on conflict antecedents. Interventions can be direct, such as creating opportunities for interaction where none were available before. Indirect interventions may be required in other situations, such as developing a protocol for information exchange between colleagues in departments that lack opportunities for communication.

Study limitation: Data collection took place in one Israeli hospital, on a small sample size. It will therefore be informative to repeat the study in other hospitals. It will be important to include participation rate which may vary across departments. A more balanced sample of nurses and physicians in the different departments will also improve the study. We anticipate that combining such studies with the methodology we developed here, based on analysis of correlation between conflict antecedents and effectiveness of alleviating tools, will help develop effective sets of alleviating tools matching range of specific disruptive behavior patterns.

## Conclusions

In conclusion, this study points to the differences in disruptive behavior patterns, causes and, consequences among hospital departments, and their impact on the choice of tools for effective intervention. The most common causes of disruptive behavior were the personalities of disputants and department leaders, miscommunication, and intense stressful working environments. The forms of disruptive behavior varied significantly between departments with some more prone to expressed behaviors such yelling and cursing, while others were more prone to hidden conflicts resulting in avoidance of interpersonal interactions. Interestingly, whether hidden or expressed, disruptive behavior is perceived to lead to negative, even dangerous effects on both patient care and staff morale and well-being and was influenced by the antecedents of disruptive behavior. These different antecedents of disruptive behavior must be addressed by different intervention tools: with some general solutions and some specific alleviating tools tailored to address directly and indirectly the unique antecedents of disruptive behavior. Therefore, applied more comprehensively and widely, the methodology presented here will help to identify and establish a set of general and specific tools for an efficient path towards resolving disruptive conflicts and can enhance the quality and cost-effectiveness of healthcare organizations.

## Additional file

Additional file 1:
**Questionnaire.**

## Abbreviations

ECU: Emergency care unit
ER: Emergency room
ICU: Intensive care unit
LD: Labor and delivery
