# Supplementary figures and images for: Universal versus tailored solutions for alleviating disruptive behavior in hospitals
Source: Isr J Health Policy Res. 2015 Sep 1;4:26. doi: 10.1186/s13584-015-0018-7 (PMC4556051; doi:10.1186/s13584-015-0018-7)

# Appendix A: Questionnaire


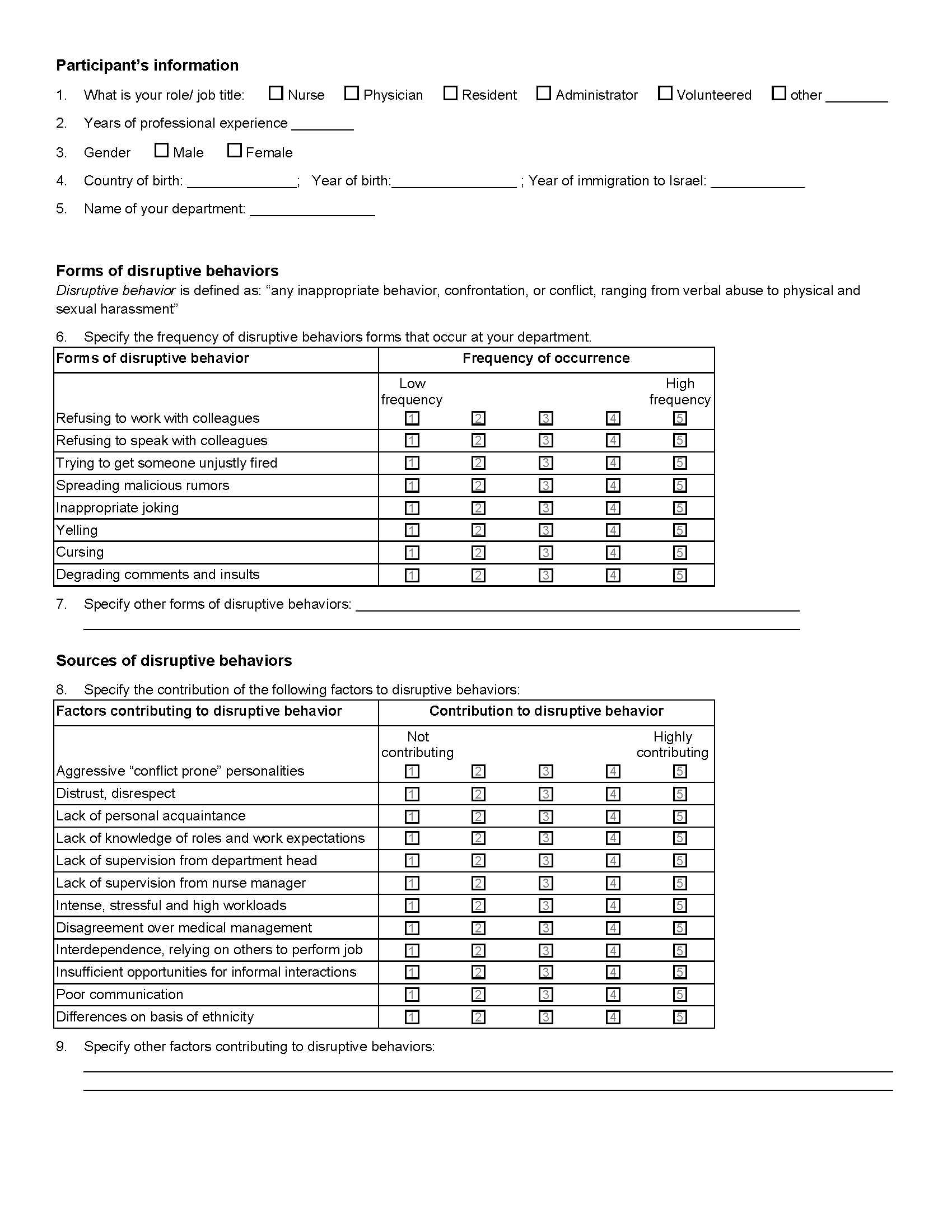


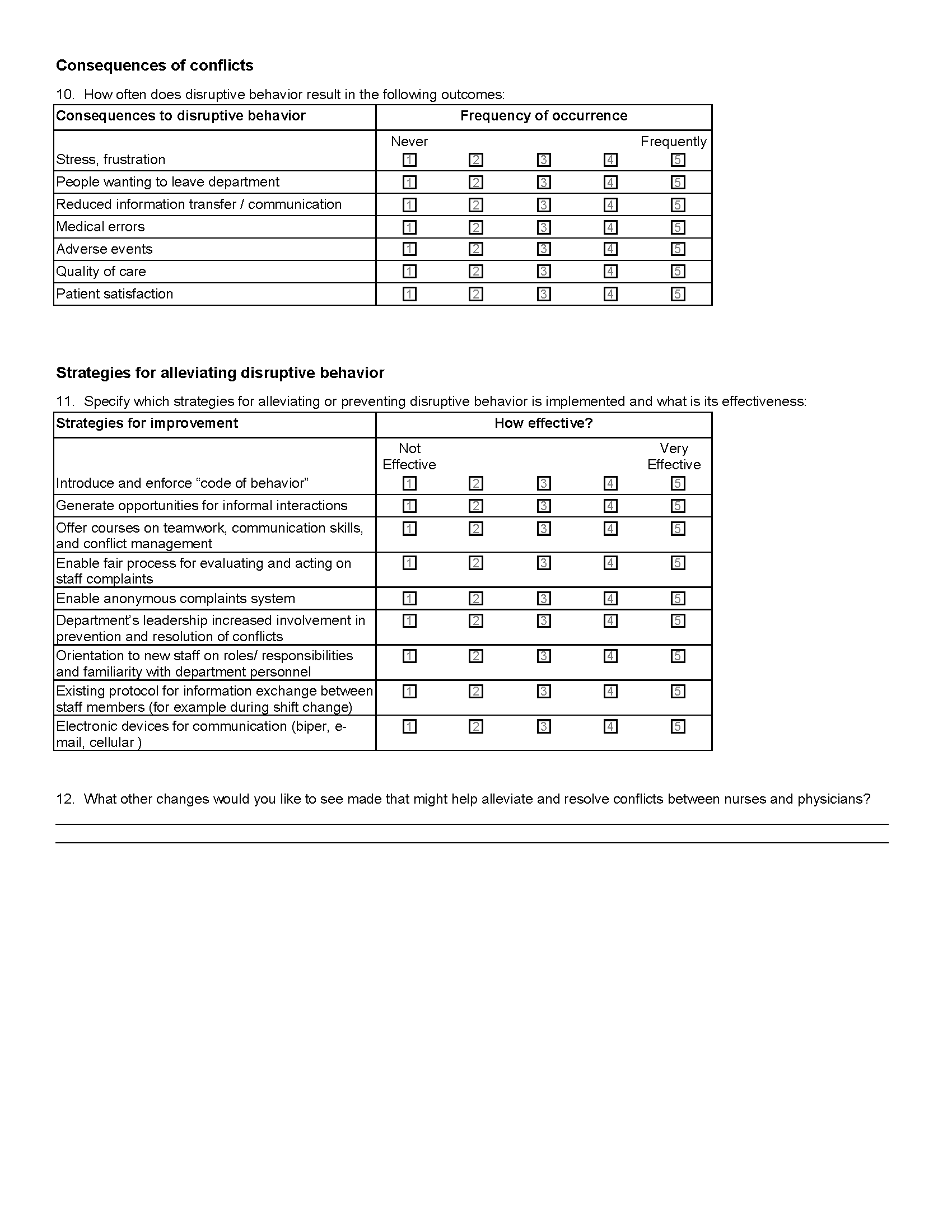

Supplement: Additional file 1: — Questionnaire. [file 13584_2015_18_MOESM1_ESM.doc]
